# Evaluation of the Effect of the First Generation iStent on Corneal Endothelial Cell Loss—A Match Case-Control Study

**DOI:** 10.3390/jcm10194410

**Published:** 2021-09-26

**Authors:** Joanna Konopińska, Emil Saeed, Łukasz Lisowski, Kinga Gołaszewska, Paweł Kraśnicki, Diana Anna Dmuchowska, Iwona Obuchowska

**Affiliations:** Department of Ophthalmology, Medical University of Bialystok, Kilinskiego 1 STR, 15-089 Bialystok, Poland; emilsaeed1986@gmail.com (E.S.); lisowski@vp.pl (Ł.L.); kin.golaszewska@gmail.com (K.G.); kraashaan@gmail.com (P.K.); diana.dmuchowska@umb.edu.pl (D.A.D.); iwonaobu@wp.pl (I.O.)

**Keywords:** corneal endothelial cell loss, intraocular pressure, minimally invasive glaucoma surgery, iStent, open-angle glaucoma, phacoemulsification with iStent

## Abstract

Glaucoma is the leading cause of irreversible blindness worldwide. The only proven factor in slowing the progression of glaucomatous neuropathy is lower intraocular pressure (IOP), which can be achieved with pharmacology, laser therapy, or surgery. However, these treatments are associated with various adverse effects, including corneal endothelial cell loss (CECL). In recent years, several novel surgeries for reducing the IOP, collectively referred to as minimally invasive glaucoma surgery (MIGS), have been developed, one of which is the iStent. However, the long-term effects of such surgeries remain unknown. We compared a group of patients with open-angle glaucoma and cataract who underwent phacoemulsification alone with a group of patients with similar demographic and clinical characteristics who underwent simultaneous phacoemulsification and iStent implantation. Overall, 26 eyes of 22 subjects who underwent a combined phacoemulsification-iStent procedure and 26 eyes of 24 subjects who underwent cataract surgery were included. Before surgery, endothelial cells accounted to 2228.65 ± 474.99 in iStent group and 2253.96 ± 404.76 in the control group (*p* = 0.836). After surgery, their number declined to 1389.77 ± 433.26 and 1475.31 ± 556.45, respectively (*p* = 0.509). There was no statistically significant difference in CECL between the two groups 18–24 months after surgery, despite increased manipulation in the anterior chamber and the presence of an implant in the trabecular meshwork in those with an iStent implant. Thus, iStent bypass implantation is a safe treatment in terms of CECL for mild-to-moderate open-angle glaucoma.

## 1. Introduction

Glaucoma is the leading cause of irreversible blindness worldwide. The only proven intervention in slowing the progression of glaucomatous neuropathy is to lower intraocular pressure (IOP), which can be achieved with pharmacotherapy, laser therapy, or surgery [1]. These treatments are associated with various adverse effects, including corneal endothelial cell loss (CECL) [2,3].

The corneal endothelium is a single layer of hexagonal-shaped cells found on the posterior surface of the cornea; its most important function is maintaining corneal transparency. Endothelial cells do not proliferate, and their numbers gradually decrease with age at 0.56% per year [3]. Endothelial cell density (ECD) is also affected by glaucoma. A significantly higher CECL has been found in eyes with open-angle glaucoma than in normal eyes [4]. In glaucoma, CECL is directly proportional to the IOP [3].

The corneal endothelium consists of a non-replicating single layer of hexagonal cells on the posterior surface of the cornea. At birth, 7500 cells/mm^2^ are found in healthy eyes, and 3000–4000 cells/mm^2^ are found in children. This amount gradually decreases, reaching at an age of 40 approximately 2500 cells/mm^2^ and at the age of 70 approximately 1500–2000 cells/mm^2^. The potential risk of corneal edema is 500–800 cells/mm^2^. The borderline density of endothelial cells is approximately 500 cells/mm^2^; below this value the cornea decompensates. The primary functions of the endothelium include the transport of nutrients from the aqueous humor to the stroma and the transport of water from the stroma to the aqueous humor, which helps to maintain the translucency of the cornea [4]. Surgical treatment of glaucoma has a risk of corneal endothelial damage due to mechanical manipulation in the anterior chamber of the eye [5]. The integrity of the endothelial cell layer is crucial as it is responsible for maintaining the translucency of the cornea and has limited regenerative capacity. Thus, damage to these cells can result in cornea clouding, edema, bullous keratopathy, and irreversible blindness [6,7,8,9,10,11,12,13,14].

In recent years, several novel surgeries for reducing the IOP, collectively referred to as minimally invasive glaucoma surgery (MIGS), have been developed. The rapid adoption of these procedures and the fact that this is still an emerging field has created the need for the data of their long-term efficacy and safety [15,16]. Safety can be a critical concern, as seen in the case of the CyPass supraciliary microshunt (Alcon Laboratories, Fort Worth, TX), which was withdrawn from the market due to its adverse effects on ECD [17]. Five years after CyPass implantation, the CECL was > 20% [10].

The use of XEN gel stent (Allergan, Belfast, Ireland) resulted in a 2.1% reduction in ECD at 3 months after surgery [18]. Two years after implantation of the XEN gel stent combined with and without phacoemulsification, CECL was comparable in both groups and was 14.3% and 14.5%, respectively [19]. Hydrus Schlemm canal microstent implantation (Ivantis, Irvine, CA, USA) during phacoemulsification resulted in a CECL of 11.7% after 6 months [20]. The 3-year data show that there is no difference in the ECD in patients operated on only for cataract and those undergoing combined surgery, i.e., phacoemulsification and Hydrus microstent implantation [20].

Another recently introduced technique for MIGS was iStent. The first-generation iStent trabecular micro-bypass stent (Glaukos, San Clemente, CA, USA) was designed to restore the natural physiological outflow of aqueous humor through the trabecular meshwork into the Schlemm’s canal. Due to its efficacy and good safety profile, this titanium microstent is increasingly being used during cataract phacoemulsification in patients with coexisting mild-to-moderate glaucoma [21,22,23,24,25,26,27]. Previous studies on the effect of iStent on the corneal endothelial status showed that one year after combined phacoemulsification-iStent surgery, the CECL ranged from 13.2% to 14.6% [21]. The 2-year follow-up showed that the ECD reduction was comparable between eyes with glaucoma in which only phacoemulsification was performed and those in which combined phacoemulsification and iStent surgery were performed, being 12.3% and 13.1%, respectively [22].

A comparison of different surgeries performed on the same patient, namely, phacoemulsification and excisional goniotomy with the Kahook Dual Blade (KDB, New World Medical, Rancho Cucamonga, CA, USA) in one eye and with iStent-phacoemulsification in the other eye, showed that 12–18 months after surgery, CECL was greater in the iStent group at 9% versus 3.4% in the Kahook Dual Blade group [28].

Previous studies about the effects of MIGS, in particular, the increasingly used iStent, on corneal endothelial status indicate that the knowledge on this topic is still inadequate, and the long-term effects of such surgeries remain unknown. The only report available to date focusing on iStent implantation during cataract surgery showed a clear distinction between the effect of the microstent itself and that of cataract phacoemulsification on ECD, although it was not a match-control study [25].

The purpose of our study was to evaluate the effect of iStent microbypass on ECD by comparing endothelial cell loss after surgery in two study groups matched according to demographic and clinical parameters. We compared a group of patients with cataract and coexisting open-angle glaucoma who underwent phacoemulsification alone with a group of patients with similar clinical characteristics who underwent simultaneous phacoemulsification and iStent implantation.

## 2. Materials and Methods

Data for the study were collected with the consent of the Bioethics Committee at the Medical University of Bialystok (consent no UMB/21/2014), in accordance with the principles of the Declaration of Helsinki and good medical practice. All patients signed written consent for the surgery, follow-up study, and use of their findings in scientific publications.

The study was designed as single-center consecutive case-control study and audit for the International Classification of Diseases, Tenth Revision (ICD-10) final diagnosis codes B18 and B19 encompassing cataract removal. Patients with open-angle glaucoma who underwent combined cataract removal by phacoemulsification with simultaneous iStent implantation or phacoemulsification alone between January 2018 and June 2019 were identified retrospectively using the Clininet medical information system. The inclusion criteria for the study were coexisting cataracts with mild-to-moderate primary open-angle glaucoma or pseudoexfoliative glaucoma. Patients with corneal endothelial dystrophy and corneal anomalies, advanced open-angle glaucoma, closed-angle glaucoma, post-inflammatory and post-traumatic glaucoma, subluxated or traumatic cataracts, and those who had undergone prior ophthalmic surgery or laser surgery were excluded. Patients with recurrent inflammation, ocular trauma, or other surgery during the follow-up period were also excluded.

Data obtained from the preoperative visit records included demographics, type, severity of glaucoma diagnosed using the standard methods [29], (Humphrey visual field analyzer, Carl Zeiss AG, Jena, Germany with SITA Standard 24-2 algorithm), lens opacification severity as determined by the Lens Opacification Classification Scale III (LOCS scale), best-corrected visual acuity (BCVA), corneal endothelial cell density (ECD), intraocular pressure (IOP), and the dose of anti-glaucoma medication used.

IOP was measured by Goldman applanation between 8 and 10 AM. The corneal endothelium was evaluated using an SP-3000P non-contact specular microscope (Topcon Medical Systems 48 Inc., Oakland, USA). The number of endothelial cells per square millimeter (cell density—CD) was measured. All tests were performed on the central cornea, several images were taken, and the image with the best resolution and quality was considered for analysis. If the image was blurry preventing the device from appropriately recognizing cell borders, the photo was repeated. Only one—the best quality image—was chosen per patient.

The same researcher (JK) manually selected the cells for analysis. Each time, 50 cells were selected if possible; if there were fewer than 50 clearly visible cells, all visible cells were selected. The camera software automatically calculated the values of the measured parameters. BCVA, IOP, ECD, and the dose of anti-glaucoma medication were analyzed during the late follow-up examination. Endothelial cell loss was defined as the difference between ECD0—ECDfinal = ECD loss.

The indication for surgery was the presence of clinically significant cataract (BCVA < 0.7, according to Snellen charts) with co-existing glaucoma with IOP ≥ 25 mmHg at the maximum number of IOP-lowering medication tolerated. The decision whether a patient would undergo cataract surgery alone or a combined procedure was made by the surgeon after counselling the patient. The main objective was to improve BCVA by replacing the lens and reducing IOP and the number of glaucoma medications.

Two groups of patients were created: the iStent group, which included patients who underwent combined phacoemulsification-iStent surgery, and the control group, which comprised patients who underwent phacoemulsification only. Groups were matched according to age, sex, follow-up period, and glaucoma type.

### 2.1. Surgical Technique

All phacoemulsification procedures in both groups were performed by the same surgeon (PK), and iStent implantation in a combined procedure was performed by another surgeon (JK).

The first generation of iStent was implanted, and the procedure was performed under topical anesthesia according to a previously described technique [30].

Briefly, after completion of phacoemulsification, carbachol was administered into the anterior chamber to constrict the pupil and improve visualization of the angle structures. Cohesive viscoelastic was administered, and an iStent was implanted through the main port created for cataract removal with an injector into the inferior nasal quadrants of the Schlemm’s canal. In all cases, one by-pass per eye was implanted. Appropriate implant fixation was confirmed by the reflux of blood into the anterior chamber. The anterior chamber was irrigated using an aspiration-irrigation system, followed by administration of cefuroxime into the anterior chamber and hydration of the ports. During phacoemulsification, following parameters were evaluated: cumulative dissipated energy (CDE), which is the amount of ultrasound energy used during surgery to remove a cataractous lens, and the aspiration time (AT).

Postoperatively, the eyes were treated with antibiotics (moxifloxacin 0.5% one drop four times a day) for 2 weeks and steroid (loteprednol 0.5% one drop, twice daily) tapered for 4 weeks. Antiglaucoma drops were discontinued at the time of surgery. They were re-initiated postoperatively if the desired IOP target was not achieved.

### 2.2. Statistical Analysis

Statistical analysis was performed using R package, version 4.0.5. Nominal variables are presented as count n (% frequency), while continuous variables are presented as mean ± SD or median (range), depending on the distribution. The normality of distribution was tested using the Shapiro–Wilk test, data skewness, and kurtosis indicators and was based on the visual assessment of histograms. The equality of variance was checked using Levene’s test. Following the matching of eyes from the control group vs. the test group, a comparison of groups was conducted using tests appropriate for dependent variables: McNemar test, paired *t*-test, or Wilcoxon signed-rank test, as appropriate. Additionally, the mean or median difference (MD) with a 95% confidence level (CI) was also calculated. A significance level of α = 0.05 was used, and all tests were two-sided.

According to the sample size calculation, the study required a sample size of 17 (number of pairs) to achieve a power of 80% and a level of significance of 5% (two sided), for detecting a mean of the differences of 75 between pairs, assuming the standard deviation of the differences to be 100.

Twenty-six eyes were matched using exact matching for age, sex, type of glaucoma, and duration of follow-up from a select group of 54 patients who underwent cataract surgery alone (control group) and from a select group of 34 patients who underwent combined surgery (iStent group).

## 3. Results

Overall, 26 eyes of 22 subjects who underwent combined phacoemulsification-iStent procedure and 26 eyes of 24 subjects who underwent cataract surgery were included in this analysis. Table 1 shows the baseline clinical characteristics of each group. The pre-matched data showed no difference in the visual field indices between the groups.

Comparison of pre- and postoperative ECD, BCVA, IOP, and the number of anti-glaucoma medications between the groups is shown in Table 2. Both baseline and final ECD levels were not significantly different between the groups. Average CECL in the iStent group was 838.88 (SD = 291.67), while in the control group it was 778.65 (SD = 423.50), *p* = 0.479. There was no significant difference in BCVA between the two groups, both at baseline and at the end of the study. The average preoperative IOP level was significantly higher in the iStent group, and the final IOP was not significantly different between the two groups.

Patients in the iStent group received more anti-glaucoma medications before surgery, most often two, compared to those in the control group, and this difference was statistically significant. At the end of the study, the number of IOP-lowering drops decreased significantly in the iStent group and became less than that in the control group. This difference was statistically significant (*p* < 0.001).

Preoperatively, mean deviations (MD) in visual field testing were −7.51 ± 7.3 dB and −8.9 ± 15.5 dB in iStent and Control groups, respectively (*p* = 0.12). After 24 months of observation, MD in groups I and II were −7.82 ± 10.4 dB and −9.1 ± 8.9 dB (*p* = 0.35), respectively. Stabilization of the visual field was observed in 83.3% and 79.7% of patients from groups I and II, respectively (*p* = 0.14). Deterioration in MD was found in 16.7% of patients in group I and 20.3% of patients in group II (*p* = 0.24).

## 4. Discussion

In this study, we compared the effect of combined surgery (iStent and phacoemulsification) vs. phacoemulsification alone on corneal endothelial cell density. We showed that there was no statistically significant difference in CECL between the two groups despite increased manipulation in the anterior chamber and the presence of an implant in the trabecular meshwork in those with an iStent implant. To our knowledge, this is the first case-control study to evaluate the effect of iStent on the endothelium. Due to the precise selection of the study groups, it was possible to eliminate the influence of factors other than the surgical procedure itself that could affect the ECD. These included sex, age, length of time between surgery and final evaluation, and type of glaucoma. ECD is significantly lower in patients with pseudoexfoliation syndrome, and the eyes of these patients are more prone to corneal endothelial decompensation after ocular surgery [25,31].

Cataract phacoemulsification is known to be associated with CECL, which is approximately 7–8% in healthy eyes 6–12 months after the surgery [5,6,7,8], 9–10% in eyes with open-angle glaucoma [7], and 14.5% in eyes with angle-closure glaucoma [8]. The following procedures cause the maximum surgical damage to the corneal endothelium: Ahmed glaucoma valve implants (New World Medical, Rancho Cucamonga, CA, USA), [11], Molteno implants (Molteno Ophthalmic Limited, Dunedin, New Zealand) [10], and Baerveldt implants (Johnson and Johnson, New Brunswick, NJ, USA) [14]. Two years after surgery, CECL was reported to range from 11.5–18.6% in the case of Ahmed valve implantation [11,14], 13.7% in the case of Baerveldt implantation [9], and 12.4% when Molteno glaucoma implants were used [10]. Trabeculectomy without mitomycin C (MMC) is associated with a CECL of 3.8–3.9% within 12 months after surgery [12]. The average CECL is 4–5.2% at two years after Ex-PRESS implantation [13], which raises concerns over its safety and use.

There are few papers published on this topic, and these usually evaluated the decrease in ECD after combined phacoemulsification-iStent surgery, without considering which procedure is responsible for the cell loss [27,32].

Only the study by Samuelson et al. [25] compared the endothelial cell loss in the phaco andiStent group with that of the cataract surgery alone group, and as in our study, there was no statistically significant difference in the decrease in ECD between the two groups. This demonstrates that iStent bypass grafting is safe for the corneal endothelium and has no or minimal effect on CECL. Mechanical manipulation in the anterior chamber associated with routine phacoemulsification is mainly responsible for CECL after conjunctival surgery. Similar findings were already described by Shiba et al. [33], who reported that standalone implantation of the first generation of the iStent has no effect on corneal endothelial status. This is the first study on this topic; however, because of the small number of eyes analyzed, it is considered a pilot study that needs confirmation.

As shown by previous studies about the effect of iStent on ECD, no significant differences were observed between the first and second generations of the iStent device. Shiba et al. [33] and Gillmann et al. [32] also used the first generation of iStent (model GTS-100), while Arriol-Villalobos et al. [27] and Samuelson et al. [25] used the second generation of iStent: GTS-400-iStent and G2-M-IS, respectively. The GTS-400 was designed to enhance trabecular outflow in the same way as with the GTS100; however, it has the advantage of easier implantation and greater reduction in IOP when performing surgery with two stents [34,35]. The different designs of the iStent injection (GTS400) from the iStent (GTS100) might result in different outcomes depending on the model used. Further studies that address the differences between the two iStent generations, especially regarding postoperative ECD and IOP, need to be conducted. Given that one GTS400 applicator contains two stents, it should only be compared to studies where two first-generation stents were implanted.

The importance of evaluating the adverse effects of MIGS is shown by the example of CyPass, implanted ab interno into the suprachoroidal space, which was withdrawn from the market by the FDA in 2018 due to the discovery of significant endothelial cell loss among patients in whom it was implanted [17].

Although the CyPass implant belongs to the new generation of micro-invasive surgery, the example shows that one cannot assume a priori that minimally invasive surgery is better than classical surgery, and studies like the current one are necessary to provide scientific evidence of its safety for the patient. As shown by more than a decade of experience using the iStent and iStent injection worldwide, this method has high safety and satisfactory results in terms of the effect in lowering the IOP and the possibility of reducing anti-glaucoma medications [28,29,30]. In a decade of iStent bypass use in the US, only 0.007% of cases required removal [25].

Our study has some limitations, including some amount of selection bias due to the specific study design. The number of subjects was small due to strict inclusion and exclusion criteria. Although a randomized controlled trial typically provides the highest quality evidence, recent statistical matching strategies can produce a highly balanced comparison of interventions utilizing extant data. In the current study, four key parameters (age, sex, glaucoma type, and follow-up period) were used to match the patients. Moreover, the researcher was not masked during the CD examination. Further prospective studies with larger numbers of patients, including those comparing different generations of iStents, are needed to determine/confirm the safety profile of this method of MIGS.

## 5. Conclusions

iStent bypass implantation is a safe treatment in the terms of effect on ECD for mild-to-moderate open-angle glaucoma. iStent implantation did not significantly increase corneal endothelial cell loss compared to phacoemulsification alone for patients who underwent surgery. Further studies assessing the effect of different types of MIGS on corneal endothelial cells number are needed.

## Figures and Tables

**Table 1:** Clinical characteristics of studied group of patients.

	iStent Group	Control Group	*P*
Sex, n (%) Male Female	6 (27.0) 16 (73.0)	9 (37.5) 15 (62.5)	>0.999
Age in years, median (range)	79 (49–83)	78 (49–87)	0.775
Type of glaucoma, n (%)			>0.999
POAG	17 (65.4)	17 (65.4)	
PXG	9 (34.6)	9 (34.6)	
Follow-up in months, mean±SD	18.50 ± 4.52	20.69 ± 4.80	0.144
LOCS III scale (NC1/NC2 /NC3)	10/12/4	11/13/2	0.865
Phacoemulsification parameters, mean ± SD			
CDE	4.9 ± 0.32	4.7 ± 0.21	0.645
AT in minutes	2.9 ± 0.31	2.7 ± 0.54	0.409

Groups were compared using the chi-square test (sex), McNemar test (glaucoma type), paired *t*-test (follow-up), or Wilcoxon signed-rank test (age); POAG, primary open-angle glaucoma; PXG, pseudoexfoliative glaucoma; LOCS III scale, lens opacification classification scale; CDE, cumulative dissipated energy; AT, aspiration time.

**Table 2:** Comparison of ECD, IOP, BCVA, and anti-glaucoma drugs between iStent and control group.

	iStent Group	Control Group	MD (95% CI)	*p*
ECD baseline ECD final	2 228.65 ± 474.99 1 389.77 ± 433.26	2 253.96 ± 404.76 1 475.31 ± 556.45	−25.31 (−274.46; 223.84) −85.54 (−348.41; 177.33)	0.836 0.509
BCVA baseline BCVA final	0.46 ± 0.25 0.85 ± 0.25	0.43 ± 0.21 0.79 ± 0.27	0.03 (−0.08; 0.15) 0.06 (−0.10; 0.22)	0.559 0.459
IOP baseline IOP final	18.04 ± 4.53 14.87 ± 3.22	15.91 ± 3.26 15.73 ± 3.72	2.13 (0.27; 3.98) −0.86 (−3.18; 1.45)	0.026 0.448
Drugs baseline ^1^ Drugs final ^1^	2.00 (0–3) 0.00 (0–2)	1.00 (1–2) 1.00 (0–2)	1.00 (0.01; 1.00) −1.00 (−1.00004; −0.99)	0.027 <0.001

Data are presented as mean ± SD or median (range) ^1^. MD, mean or median1 difference (iStent minus control) with 95% confidence interval (CI); ECD, endothelial cell loss; BCVA, best-corrected visual acuity; and IOP, intraocular pressure.

## Data Availability

All the materials and information will be available upon an e-mail request to the corresponding author. Names and exact data of the participants of the study may not be available owing to patient confidentiality and privacy policy.
